# HIV and incarceration: prisons and detention

**DOI:** 10.1186/1758-2652-14-26

**Published:** 2011-05-19

**Authors:** Ralf Jürgens, Manfred Nowak, Marcus Day

**Affiliations:** 1 97 de Koninck, Mille-Isles, Quebec, J0R 1A0, Canada; 2 University Vienna; Director, Ludwig Boltzmann Institute of Human Rights, Vienna; UN Special Rapporteur on Torture; Ludwig Boltzmann Institute of Human Rights, Freyung 6/2, 1010 Vienna, Austria; 3 Caribbean Drug & Alcohol Research Institute, Box 1419, Castries, Saint Lucia

## Abstract

The high prevalence of HIV infection among prisoners and pre-trial detainees, combined with overcrowding and sub-standard living conditions sometimes amounting to inhuman or degrading treatment in violation of international law, make prisons and other detention centres a high risk environment for the transmission of HIV. Ultimately, this contributes to HIV epidemics in the communities to which prisoners return upon their release.

We reviewed the evidence regarding HIV prevalence, risk behaviours and transmission in prisons. We also reviewed evidence of the effectiveness of interventions and approaches to reduce the risk behaviours and, consequently, HIV transmission in prisons.

A large number of studies report high levels of risk behaviour in prisons, and HIV transmission has been documented. There is a large body of evidence from countries around the world of what prison systems can do to prevent HIV transmission. In particular, condom distribution programmes, accompanied by measures to prevent the occurrence of rape and other forms of non-consensual sex, needle and syringe programmes and opioid substitution therapies, have proven effective at reducing HIV risk behaviours in a wide range of prison environments without resulting in negative consequences for the health of prison staff or prisoners.

The introduction of these programmes in prisons is therefore warranted as part of comprehensive programmes to address HIV in prisons, including HIV education, voluntary HIV testing and counselling, and provision of antiretroviral treatment for HIV-positive prisoners. In addition, however, action to reduce overcrowding and improve conditions in detention is urgently needed.

## Review

### Forgotten prisoners: a global crisis of conditions in detention

A global crisis of conditions in detention is being witnessed by the United Nations Special Rapporteur on Torture and Other Forms of Cruel, Inhuman or Degrading Treatment or Punishment. The Special Rapporteur exercises a mandate entrusted to him by the highest human rights body of the United Nations (UN), the Human Rights Council, to investigate the situation of torture and ill-treatment in all countries of the world. He presents reports about his findings and recommendations to the General Assembly in New York and the Human Rights Council in Geneva.

In addition to conducting research and dealing with a high number of individual complaints, since 2005, he has carried out fact-finding missions to roughly 20 countries in all regions of the world, among them Georgia, Mongolia, China (including the autonomous regions of Tibet and Qinjang), Nepal, Jordan, Paraguay, Togo, Nigeria, Sri Lanka, Indonesia, Denmark (including Greenland), the Republic of Moldova (including Transnistria), Equatorial Guinea, Uruguay, Kazakhstan, Jamaica and Papua New Guinea.

Since torture usually takes place behind closed doors, the Special Rapporteur spends a significant amount of time during the fact-finding missions in prisons, remand centres, police lock-ups, psychiatric institutions, and special detention facilities for women, children, asylum seekers, migrants and people who use drugs. By assessing conditions of detention in each country he visits, the UN Special Rapporteur on Torture also acts as a *de facto *special rapporteur on prison conditions.

Governments have no legal obligation to invite the UN Special Rapporteur to their countries, and several governments, notably in the Middle East, have refused investigations into torture and ill-treatment in their countries. Others have invited the Rapporteur and later cancelled or "postponed" their invitations, often at the last minute: the USA (in respect of the detention facilities in Guantánamo Bay), the Russian Federation, Zimbabwe and Cuba. Sometimes, the strict terms of reference are the reason for the reluctance of states to receive the Special Rapporteur. These terms include the possibility of: carrying out unannounced visits to places of detention; bringing a team of experts into prisons, including a forensic expert, with the necessary equipment to document torture and ill-treatment (e.g., photo and video cameras); and conducting private (unsupervised) interviews with detainees [1].

The conclusions of the UN Special Rapporteur are alarming: with very few exceptions (such as Denmark and Greenland) [2], torture in detention facilities is practiced in most of the countries he has visited, often in a routine, widespread or even systematic manner, such as in Nepal [3] and Equatorial Guinea [4]. In some countries, including Sri Lanka [5] and Jordan [6], the Special Rapporteur observed that the methods of torture used are simply shocking and remind one of times forgotten.

But for most of the detainees interviewed by the UN Special Rapporteur, their experience of torture during the first days or weeks of police custody aimed at extracting a confession or information was little compared with the continued suffering of detainees. They had endured this suffering during many months of police custody with no more than a place to sit on the dirty floor (e.g., in Equatorial Guinea, Jamaica [7] and Papua New Guinea [8], during many years of pre-trial detention virtually forgotten by prosecutors, judges and the outside world (e.g., in Nigeria [9], Paraguay [10] and Uruguay [11]), and during decades of incarceration in overcrowded prisons, often in isolation or under intolerable conditions on death row and similar strict confinement for long-term prisoners (e.g., Mongolia [12], Georgia [13] and Moldova [14]).

In China, an unbearable pressure of re-education and brainwashing is exerted on the entire prison population, ranging from special re-education through labour camps to remand centres and correctional institutions, until the will and dignity of the person concerned is finally broken [15]. In his 2009 report to the UN General Assembly, the Special Rapporteur concluded that "in many countries of the world, places of detention are constantly overcrowded, and filthy locations, where tuberculosis and other highly contagious diseases are rife [, ...] lack the minimum facilities necessary to allow for a dignified existence [16]."

In other words, conditions of detention in many countries amount to inhuman or degrading treatment in violation of international law. There is a veritable global crisis of conditions of detention. Without understanding this background, it is not possible to appreciate the challenges posed by HIV in detention, to which we now turn.

### Two epidemics: HIV and incarceration

HIV hit prisons early and it hit them hard. The rates of HIV infection among prisoners in many countries are significantly higher than those in the general population. Coincident with the HIV/AIDS epidemic, many countries have been experiencing a significant increase in the incarcerated population, often as a result of an intensification of the enforcement of drug laws in an effort to limit the supply and use of illegal drugs. Each of the two "epidemics" - HIV and incarceration - has affected the other.

For the purposes of this paper, the term, "prisoner", is used broadly to refer to adult and juvenile males and females detained in criminal justice and correctional facilities: during the investigation of a crime; while awaiting trial; after conviction and before sentencing; and after sentencing. Although the term does not formally cover persons detained for reasons relating to immigration or refugee status, and those detained without charge, most of the considerations in this paper apply to them, as well. The term, "prison", is used to refer to all criminal justice and correctional facilities.

#### The HIV epidemic in prisons

HIV surveillance has been the most common form of HIV research in prison, although this has largely been restricted to high-income countries. Data from low- and middle-income countries are more limited [17]. Even within high-income countries, the precise number of prisoners living with HIV is difficult to estimate. Rates of HIV infection reported from studies undertaken in a single prison or region may not accurately reflect HIV prevalence in prisons across the country. 

Nevertheless, reviews of HIV prevalence in prison have shown that HIV infection is a serious problem, and one that requires immediate action [18]. In most countries, HIV prevalence rates in prison are several times higher than in the community outside prisons, and this is closely related to the rate of HIV infection among people who inject drugs in the community and the proportion of prisoners convicted for drug-related offences [19]. In other countries, particularly in sub-Saharan Africa, elevated HIV prevalence rates in prisons reflect the high HIV prevalence rates in the general population [20]. Everywhere, the prison population consists of individuals with greater risk factors for contracting HIV (and HCV and TB) compared with the general population outside of prisons. Such characteristics include injecting drug use, poverty, alcohol abuse, and living in minority communities with reduced access to healthcare services [21].

Studies have shown HIV prevalence that ranges from zero in a young male offenders institution in Scotland [22] and among prisoners in Iowa, United States, in 1986 [23], to 33.6% in an adult prison in Catalonia, Spain [24], to more than 50% in a correctional facility for women in New York City [25]. As early as 1988, about half of the prisoners in Madrid [26] and 20% of prisoners in New York City tested HIV positive [27]. More recent reports show that HIV prevalence rates remain high in prisons in North America [28-30] and western Europe, although they have decreased in countries like Spain that have introduced comprehensive HIV interventions in prisons, including needle and syringe programmes and methadone maintenance treatment [31].

In the countries of central and eastern Europe and the former Soviet Union, HIV prevalence is particularly high in prisons in Russia and Ukraine, but also in Lithuania, Latvia and Estonia. In Russia, by late 2002, the registered number of people living with HIV/AIDS in the penal system exceeded 36,000, representing approximately 20% of known HIV cases. In Latin America, prevalence among prisoners in Brazil and Argentina was reported to be particularly high, with studies showing rates of between 3% and more than 20% in Brazil and from 4% to 10% in Argentina.

Rates reported from studies in other countries, including Mexico, Honduras, Nicaragua and Panama are also high [32]. In India, one study found that the rates were highest among female prisoners, at 9.5% [33]. In Africa, a study undertaken in Zambia found a rate of 27% [34]. The highest HIV prevalence reported among a national prison population was in South Africa, where estimates put the figure as high as 41.4% [32]. Conversely, some countries report zero prevalence; most of these are in north Africa or the Middle East [32].

The HIV epidemic in prisons is not occurring alone: prevalence rates of viral hepatitis in prisons are even higher than HIV rates [35,36]. In particular, while the World Health Organization (WHO) estimates that about 3% of the world's population has been infected with the hepatitis C virus (HCV), [37] estimates of the prevalence of HCV in prisons range from 4.8% in an Indian jail [38] to 92% in two prisons in northern Spain [39,40].

Tuberculosis (TB) is also common: in some countries, it has been estimated that it is 100 times more common in prisons than in the community [41]. Wherever TB is evident in prisons, it is a significant health problem. Sub-standard prison living conditions, including overcrowding, poor ventilation, poor lighting and inadequate nutrition, make the attempts to control the spread of TB in prisons more difficult. TB incidence rates are therefore very high in many prisons. Moreover, prisons in geographically disparate places (from Thailand to New York State to Russia) have reported high levels of drug-resistant TB. TB poses a substantial danger to the health of all prisoners, staff and the community outside prisons. Prisoners living with HIV are at particular risk. HIV infection is the most important risk factor for the development of TB, and TB is the main cause of death among people living with HIV. TB mortality in prisons is elevated [42].

Within prison populations, certain groups have higher levels of infection. In particular, the prevalence of HIV and HCV infection among women tends to be higher than among men [18].

#### The epidemic of incarceration

Coincident with the emergence of HIV and later HCV, many countries have been experiencing a significant increase in the size of their incarcerated population. As of 1998, more than 8 million people were held in penal institutions throughout the world, either as pre-trial detainees or having been convicted and sentenced. As of December 2008, more than 9.8 million people were incarcerated [43]. If prisoners in "administrative detention" in China are included, the total was more than 10.6 million. Between 2005 and 2008, prison populations rose in 71% of countries [43]. Each year, some 30 million people enter and leave prison establishments.

The USA has the highest prison population rate in the world (748 per 100,000 of the national population), followed by Russia (595), Rwanda (593) and a number of countries in eastern Europe and in the Caribbean. Countries with particularly low rates include Liechtenstein (28), Nepal (24), Nigeria (29) and India (32). On average, the prison population rate is 145 per 100,000. Certain regions, such as the Caribbean, eastern Europe, central Asia and southern Africa, have much higher rates, while others, such as northern and western Europe, western Africa and Oceania (with few exceptions) have much lower average rates [43].

In the absence of internationally agreed minimum space requirements for detainees, it is difficult to measure the level of overcrowding, but overcrowding is a common problem. The best proxy indicator is the official occupancy rate, i.e., the percentage of the actual number of prisoners in relation to the official maximum capacity of the prison system as a whole. Although states can easily manipulate these statistics by simply enlarging the official maximum capacity, some 60% of all countries in the world report an occupancy rate of more than 100%, which means that they hold more prisoners than the maximum capacity. In 16 countries, primarily in Africa, the occupancy rate exceeds 200% [44].

There are various reasons for such extreme overcrowding, including, above all: the lack of non-custodial measures for dealing with crime, (i.e., incarceration is regarded as the only measure for dealing with suspected criminals rather than as a measure of last resort); the criminalization of behaviours seen as socially undesirable by many legislators (sex work, drug-related offences, homosexuality, etc.); corruption; and the non-functioning of the criminal justice system in many countries.

The best indicator for the failure of the criminal justice system is the percentage of pre-trial detainees compared with the total prison population. According to international law, pre-trial detention should be the exception and is only permissible for the shortest period of time (usually no longer than a few months) [45]. In reality, persons suspected of petty and other criminal offences who lack money for bribes or bail often spend many years in pre-trial detention, forgotten by prosecutors and judges. In many countries in Africa (Liberia, Mali, Benin, Niger, Congo Brazzaville, Nigeria, Burundi and Cameroon), Latin America (Haiti, Bolivia, Paraguay, Honduras and Uruguay), and Asia (Bangladesh, India, Pakistan and the Philippines), pre-trial detainees comprise more than 60% of the total prison population. It is, therefore, not surprising that high occupancy rates and pre-trial detention rates correlate in many countries, such as in Haiti before the earthquake in January 2010, Benin, Bangladesh, Burundi, Pakistan and Mali [43].

In many parts of the world, the growth in prison populations (and often the resulting increase in overcrowding) has been the result of an intensification of the enforcement of drug laws in an effort to limit the supply and use of illegal drugs. As a result of the large number of prisoners convicted for drug-related offences, the demographic and epidemiological characteristics of the incarcerated population are significantly different today in many countries from what they were two decades ago. Consistent with the nature of the crimes for which they are convicted, incarcerated individuals have a high prevalence of drug dependence, mental illness and infectious diseases, including HIV [46].

By choosing mass imprisonment as the main response to the use of drugs, countries have created a *de facto *policy of incarcerating more and more individuals with HIV infection [47]. Many prisoners serve short sentences, and recidivism to prison is common. Consequently, HIV-positive people (and at-risk individuals) move frequently between prisons and their home communities. For example, in the Russian Federation, each year, 300,000 prisoners, many of whom are living with HIV, viral hepatitis and/or TB, are released from prisons [48]. Most prisoners will return to their home communities within a few years. The high degree of mobility between prison and community means that communicable diseases and related illnesses transmitted or exacerbated in prison do not remain there. When people living with HIV and HCV (and/or TB) are released from incarceration, prison health issues necessarily become community health issues.

### Risk behaviours in prison

#### Injecting drug use

For people who inject drugs, imprisonment is a common event, with studies from a large number of countries reporting that between 56% and 90% of people who inject drugs had been imprisoned at some stage [49,50]. Multiple prison sentences are more common for prisoners who inject drugs than for other prisoners [51].

Some people who used drugs prior to imprisonment discontinue their drug use while in prison. However, many carry on using on the inside, often with reduced frequency and amounts [51], but sometimes maintaining the same level of use [52]. Prison is also a place where drug use is initiated, often as a means to release tension and to cope with being in an overcrowded and often violent environment [53,54].

Injecting drug use in prison is of particular concern given the potential for transmission of HIV, TB and viral hepatitis. Those who inject drugs in prisons often share needles and syringes and other injecting equipment, which is an efficient way of transmitting HIV [55]. A large number of studies from countries around the world report high levels of injecting drug use, including among female prisoners [56,57]. Although more research has been carried out on injecting drug use in prisons in high-income countries, studies from low-income and middle-income countries have found similar results. In Iran, for example, about 10% of prisoners are believed to inject drugs, and more than 95% of them are reported to share needles [58]. Injecting drug use has also been documented in prisons in countries in eastern Europe and central Asia [59-62], and there are also reports of injecting drug use in prisons in Latin America [63] and sub-Saharan Africa [64].

#### Consensual and non-consensual sexual activity

It is challenging to obtain reliable data on the prevalence of sexual activities in prisons because of the many methodological, logistical and ethical challenges of undertaking a study of sexual activity in prisons. Sex, with the exception of authorized conjugal visits, violates prison regulations. Many prisoners decline to participate in studies because they claim not to have engaged in any high-risk behaviour [65]. Prisoners who do participate may be too embarrassed to admit to engaging in same-sex sexual activity for fear of being labelled as weak or gay, and they may fear punitive measures.

Despite these challenges, studies undertaken in a large number of countries show that consensual and non-consensual sex does occur in prisons. Estimates of the proportion of prisoners who engage in consensual same-sex sexual activity in prison vary widely, with some studies reporting relatively low rates of 1% to 2% [66,67], while other studies report rates between 4% and 10% [59,68-70] or higher [71], particularly among female prisoners [56,72].

Some same-sex sexual activity occurs as a consequence of sexual orientation. However, most men who have sex in prisons do not identify themselves as homosexuals and may not have experienced same-sex sex prior to their incarceration [73].

Distinguishing coerced sex from consensual sex in prison can be difficult: prisoner sexual violence is a complex continuum that includes a host of sexually coercive (non-consensual) behaviours, including sexual harassment, sexual extortion and sexual assault. It can involve prisoners and/or staff as perpetrators. Rape in prison can be unimaginably vicious and brutal. Gang assaults are not uncommon, and victims may be left beaten, bloody and, in the most extreme cases, dead [18]. Yet overtly violent rapes are only the most visible and dramatic form of sexual abuse behind bars. Many victims of sexual violence in prison may have never been explicitly threatened, but they have nonetheless engaged in sexual acts against their will, believing they had no choice [74].

Most studies on incidence of sexual violence in prison have focused on male victims in the United States, typically reporting high rates of "sexual aggression" (11% to 40%), while reporting lower rates of "completed rape" of usually between 1% and 3% [18]. Lower levels of sexual violence than in the United States have been reported in some other developed countries. International prison research has revealed that sexual violence occurs in prisons around the world [74,75].

In prisons, with the exception of countries in which injecting drug use is rare, sexual activity is considered to be a less significant risk factor for HIV transmission than sharing of injecting equipment. Nevertheless, sexual activities can place prisoners at risk of contracting HIV and other sexually transmitted infections (STIs). Violent forms of unprotected anal or vaginal intercourse, including rape, carry the highest risk of HIV transmission [76]. Environmental or population conditions or factors that affect the risk of HIV and other STI transmission through sexual activity in prison include: the prevalence of infection in the particular prison or sub-section of the prison; the prevalence of various forms of sexual activity; and whether commodities, such as condoms, lubricant and dental dams, are provided and accessible to prisoners.

#### Other risk factors

Additional risk factors for blood-borne infections include the sharing or re-use of tattooing and body piercing equipments, sharing of razors for shaving, blood-sharing/"brotherhood" rituals and the improper sterilization or re-use of medical or dental instruments.

Factors related to the prison infrastructure and prison management contribute indirectly to vulnerability to HIV and other infections. They include overcrowding, violence, gang activities, lack of protection for vulnerable or young prisoners, prison staff that lack training or may be corrupt, and poor medical and social services

### HIV transmission resulting from risk behaviours in prisons

The prevalence of risk behaviours, coupled with the lack of access to prevention measures in many prisons, can result in frighteningly quick spread of HIV. There were early indications that extensive HIV transmission could occur in prisons. In Thailand, the first epidemic outbreak of HIV in the country likely began among people who inject drugs in the Bangkok prison system in 1988 [77]. Since then, a large number of studies from countries in many regions of the world have reported HIV and/or HCV seroconversion within prisons or shown that a history of imprisonment is associated with prevalent and incident HIV and/or HCV and/or hepatitis B virus (HBV) infection among people who inject drugs [18].

HIV infection has been significantly associated with a history of imprisonment in countries in western and southern Europe (including among female prisoners [78-83]), but also in Russia [84], Canada [85], Brazil [86], Iran [87] and Thailand [88]. Using non-sterile injecting equipment in prison was found to be the most important independent determinant of HIV infection in a number of studies [18].

The strongest evidence of extensive HIV transmission through injecting drug use in prison has emerged from documented outbreaks in Scotland [89], Australia [90], Russia [91] and Lithuania [92]. Outbreaks of HIV have also been reported from other countries [93].

Well-documented evidence exists for STI intra-prison transmission through sexual contacts among prisoners, for example in Russia and in Malawi [91,73]. Evidence also exists of HIV intra-prison transmission through sexual contacts among prisoners. In one United States study of HIV transmission in prison, sex between men accounted for the largest proportion of prisoners who contracted HIV inside prison [94].

### International human rights and the responsibility of prison systems

By its very nature, imprisonment involves the loss of the right to liberty. However, prisoners retain their other rights and privileges, except those necessarily removed or restricted by the fact of their incarceration. In particular, prisoners, as every other person, have a right to the highest attainable level of physical and mental health: the state's duty with respect to health does not end at the gates of prisons [95].

The failure to provide prisoners with access to essential HIV prevention measures and to treatment equivalent to that available outside is a violation of prisoners' right to health in international law. Moreover, it is inconsistent with international instruments that deal with rights of prisoners, prison health services and HIV/AIDS in prisons, including the United Nations' Basic Principles for the Treatment of Prisoners [96], the WHO Guidelines on HIV Infection and AIDS in Prisons [97], and the International Guidelines on HIV/AIDS and Human Rights [98].

According to the WHO guidelines, "[a]ll prisoners have the right to receive health care, including preventive measures, equivalent to that available in the community without discrimination, in particular with respect to their legal status or nationality" [97].

The International Guidelines on HIV/AIDS and Human Rights identifies the following specific action in relation to prisons [98]:

Prison authorities should take all necessary measures, including adequate staffing, effective surveillance and appropriate disciplinary measures, to protect prisoners from rape, sexual violence and coercion. Prison authorities should also provide prisoners (and prison staff, as appropriate), with access to HIV-related prevention information, education, voluntary testing and counselling, means of prevention (condoms, bleach and clean injection equipment), treatment and care and voluntary participation in HIV-related clinical trials, as well as ensure confidentiality, and should prohibit mandatory testing, segregation and denial of access to prison facilities, privileges and release programmes for HIV-positive prisoners. Compassionate early release of prisoners living with AIDS should be considered.

### Preventing and responding to HIV and other infections in prisons: a human rights and public health imperative

Two elements are key to preventing and responding to HIV and other infections, such as hepatitis B and C and TB, in prisons:

• Introducing comprehensive prevention measures

• Providing treatment, care and support, including antiretroviral treatment for HIV, and ensuring continuity of care between prisons and the community.

In addition, improving prison conditions and undertaking other prison reforms and reducing prison populations is also essential.

### Introducing comprehensive prevention measures

#### Information and education

Education is an essential precondition to the implementation of HIV prevention measures in prisons. The World Health Organization's Guidelines on HIV Infection and AIDS in Prisons recommends that both prisoners and prison staff be informed about ways to prevent HIV transmission [97]. Written materials should be appropriate for the educational level in the prison population. Furthermore, prisoners and staff should participate in the development of educational materials. Finally, peer educators can play a vital role in educating other prisoners.

However, information and education alone are not sufficient responses to HIV in prisons. A few evaluations have indicated improvements in levels of knowledge and self-reported behavioural change as a result of prison-based educational initiatives [18]. But education and counselling are not of much use to prisoners if they do not have the means (such as condoms and clean injecting equipment) to act on the information provided.

#### HIV testing and counselling

HIV testing and counselling (HTC) is important for two reasons: as part of an HIV prevention programme (it gives those who may be engaging in risky behaviours information and support for behaviour change); and as a way to diagnose those living with HIV and offer them appropriate treatment, care and support.

In practice, HTC in prisons is often available only on demand of prisoners, but in some systems, HTC is easily available. In some other systems, HTC is undertaken routinely or is even compulsory. There is evidence suggesting that mandatory HIV testing and segregation of HIV-positive prisoners is costly, inefficient and can have negative health consequences for segregated prisoners [18].

Consistent with HTC guidance developed for prisoners [99], detainees and people undergoing compulsory drug treatment, countries should ensure that all people in these settings have easy access to HTC programmes at any time during their stay. They should be informed about the availability of services, both at the time of their admission and regularly thereafter. In addition, healthcare providers in these settings should offer HTC to all during medical examinations, and recommend HTC in the event of signs, symptoms or medical conditions that could indicate HIV infection, including TB, to assure appropriate diagnosis and access to necessary HIV treatment, care and support as indicated. Efforts to increase access to HTC should not be undertaken in isolation, but as part of comprehensive HIV programmes aimed at improving healthcare, decreasing stigma and discrimination, protecting confidentiality of medical information, and vastly scaling up access to comprehensive HIV prevention, treatment, care and support.

All forms of coercion must be avoided and HIV testing must always be done with informed consent, adequate pre-test information or counselling, post-test counselling, protection of confidentiality, and referral to services.

#### Provision of condoms and prevention of rape, sexual violence and coercion

Recognizing the fact that sex occurs in prisons and given the risk of disease transmission that it carries, providing condoms has been widely recommended. As early as 1991, 23 of 52 prison systems surveyed by the World Health Organization provided condoms to prisoners [100]. Today, many more prison systems make condoms available, including most systems in western Europe, Canada and Australia, some prisons in the United States, parts of eastern Europe and central Asia, and countries like Brazil, South Africa, Iran and Indonesia [101].

There is evidence that condoms can be provided in a wide range of prison settings - including in countries in which same-sex activity is criminalized - and that prisoners use condoms to prevent HIV infection during sexual activity when condoms are easily accessible in prison (i.e., when prisoners can pick them up confidentially, without having to ask for them) [101]. No prison system allowing condoms has reversed its policy, and none has reported security problems or any other relevant major negative consequences. In particular, it has been found that condom access represents no threat to security or operations, does not lead to an increase in sexual activity, and is accepted by most prisoners and correctional officers once it is introduced [101].

However, in some countries where legal sanctions against sodomy exist in the community outside prison, and where there are deeply held beliefs and prejudices against homosexuality, introduction of condoms into prisons as an HIV prevention measure may have to be particularly well prepared. This can be done through education and information about the purpose of the introduction of condoms, as well as initiatives to counter the stigma that people engaging in same-sex activity face.

Finally, while providing condoms in prisons is important, it is not enough to address the risk of sexual transmission of HIV. Violence, including sexual abuse, is common in many prison systems. In many prison systems, HIV prevention depends as much or more on prison and penal reform than on condoms. Prison and penal reform need to greatly reduce the prison populations so that the few and often underpaid guards are able to protect the vulnerable prisoners from violence - and sexual coercion.

The Guidelines on HIV Infection and AIDS in Prisons [97] and the International Guidelines on HIV/AIDS and Human Rights [98] highlight the reality that prison authorities are responsible for combating aggressive sexual behaviour, such as rape, exploitation of vulnerable prisoners and all forms of prisoner victimization by providing adequate staffing, effective surveillance, disciplinary sanctions, and education, work and leisure programmes. Structural interventions, such as better lighting, shower and sleeping arrangements, are also needed.

Conjugal visits should also be allowed and an appropriate section of the prison outfitted for this purpose. Condoms should be available in that section, and prisoners should be allowed to carry condoms back to the main prison, thus allowing for further discreet distribution.

#### Needle and syringe programmes

The first prison needle and syringe programme (NSP) was established in Switzerland in 1992. Since then, NSPs have been introduced in more than 60 prisons in 11 countries in Europe and central Asia. In some countries, only a few prisons have NSPs. However, in Kyrgyzstan and Spain, NSPs have been rapidly scaled up and operate in a large number of prisons [102].

Germany is the only country in which prison NSPs have been closed. At the end of 2000, NSPs had been successfully introduced in seven prisons, and other prisons were considering implementing them. However, since that time, six of the programmes have been closed as a result of political decisions by the newly elected conservative state governments, without consultation with prison staff. Since the programmes closed, prisoners have gone back to sharing injecting equipment and to hiding it, increasing the likelihood of transmission of HIV and HCV [103]. Staff have been among the most vocal critics of the governments' decision to close down the programmes, and have lobbied the governments to reinstate the programmes [103].

In most countries with prison NSPs, implementation has not required changes to laws or regulations in order to allow it. Across the 11 countries, various models for the distribution of sterile injecting equipment have been used, including anonymous syringe dispensing machines, hand-to-hand distribution by prison health staff and/or non-government organization workers, and distribution by prisoners trained as peer outreach workers [102].

Systematic evaluations of the effects of NSPs on HIV-related risk behaviours and of their overall effectiveness in prisons have been undertaken in 10 projects. These evaluations and other reports demonstrate that NSPs are feasible in a wide range of prison settings, including in men and women's prisons, prisons of all security levels, and small and large prisons. Providing sterile needles and syringes is readily accepted by people who inject in prisons and contributes to a significant reduction of syringe sharing over time. It also appears to be effective in reducing resulting HIV infections [102].

At the same time, there is no evidence to suggest that prison-based NSPs have serious, unintended negative consequences. In particular, they do not lead to increased drug use or injecting; nor are they used as weapons [102]. Evaluations have found that NSPs in prisons actually facilitate referral of people who use drugs to drug dependence treatment programmes [104,105].

Studies have shown that important factors in the success of prison NSPs include easy and confidential access to the service, providing the right type of syringes and building trust with the prisoners accessing the programme [102]. For example, in Moldova, only a small number of prisoners accessed the NSP when it was located within the healthcare section of the prison. It was only when prisoners could obtain sterile injecting equipment from fellow prisoners, trained to provide harm-reduction services, that the amount of equipment distributed increased significantly [106].

Following an exhaustive review of the international evidence, WHO, the United Nations Office on Drugs and Crime (UNODC) and the Joint United Nations Programme on HIV/AIDS (UNAIDS) in 2007 recommended that "prison authorities in countries experiencing or threatened by an epidemic of HIV infections among people who inject drugs should introduce and scale up NSPs urgently" [102].

#### Bleach programmes

Programmes providing bleach or other disinfectants for sterilizing needles and syringes to reduce HIV transmission among people who inject drugs in the community were first introduced in San Francisco, United States, in 1986 [107]. Such programmes have received support, particularly in situations where opposition to NSPs in the community or in prisons has been strongest.

The number of prison systems that make bleach or other disinfectants available to prisoners has continued to grow, but already in 1991, 16 of 52 prison systems surveyed made them available, including in Africa and central America [100]. Today, bleach or other disinfectants are available in many prison systems, including in Australia, Canada, Indonesia, Iran and some systems in eastern Europe and central Asia [102].

Evaluations of bleach programmes in prisons have shown that distribution of bleach or other disinfectants is feasible and does not compromise security [102]. However, WHO has concluded that the "evidence supporting the effectiveness of bleach in decontamination of injecting equipment and other forms of disinfection is weak" [108]. While the efficacy of bleach as a disinfectant for inactivating HIV has been shown in laboratory studies, field studies have cast "considerable doubt on the likelihood that these measures could ever be effective in operational conditions" [108]. Moreover, studies assessing the effect of bleach on HCV prevalence did not find a significant effect of bleach on HCV seroconversion [109,110].

For these reasons, bleach programmes are inadequate to address the risks associated with sharing of injecting equipment and are regarded as a second-line strategy to NSPs. WHO, UNODC and UNAIDS have recommended that bleach programmes be made available in prisons where "authorities continue to oppose the introduction of NSPs despite evidence of their effectiveness, and to complement NSPs" [102].

#### Opioid substitution therapy and other drug dependence treatment

Since the early 1990s, and mostly in response to raising HIV rates among people who inject drugs in the community and in prison, there has been a marked increase in the number of prison systems providing opioid substitution therapy (OST) to prisoners. Today, prison systems in nearly 40 countries offer OST to prisoners, including most systems in Canada and Australia, some systems in the United States, and most of the systems in the 15 "old" European Union (EU) member states [111], as well as Iran, Indonesia and Malaysia [112]. In Spain, according to 2009 data, 12% of all prisoners received OST [112]. However, in most other prison systems, coverage is much lower.

OST programmes are also provided in some of the states that joined the EU more recently (including Hungary, Malta, Slovenia and Poland), although they often remain small and benefit only a small number of prisoners in need [113]. A small number of systems in eastern Europe and central Asia have also started OST programmes (such as Moldova and Albania) or are planning to do so soon [113].

Reflecting the situation in the community, most prison systems make OST available in the form of methadone maintenance treatment (MMT). Buprenorphine maintenance treatment is available only in a small number of systems, including in Australia and some European countries [114,115].

Generally, drug-free treatment approaches continue to dominate interventions in prisons in most countries [116]. OST remains controversial in many prison systems, even in countries where it accepted as an effective intervention for opioid dependence in the community outside of prisons. Prison administrators have often not been receptive to providing OST due to philosophical opposition to this type of treatment and concerns about whether the provision of such therapy will lead to diversion of medication, violence and/or security breaches [117].

A recent comprehensive review showed that OST, in particular with MMT, is feasible in a wide range of prison settings [113]. As is the case with OST programmes outside prisons, those inside prisons are effective in reducing the frequency of injecting drug use and associated sharing of injecting equipment if a sufficient dosage is provided (more than 60 mg per day) and treatment is provided for longer periods of time (more than six months) or even for the duration of incarceration [118].

In addition, evaluations of prison-based MMT found other benefits, both for the health of prisoners participating in the programmes, and for prison systems and the community. For example, re-incarceration is less likely among prisoners who receive adequate OST, and OST has been shown to have a positive effect on institutional behaviour by reducing drug-seeking behaviour and thus improving prison safety [113]. While prison administrations have often initially raised concerns about security, violent behaviour and diversion of methadone, these problems have not emerged or have been addressed successfully where OST programmes have been implemented [113].

WHO, UNODC and UNAIDS have recommended that "prison authorities in countries in which OST is available in the community should introduce OST programmes urgently and expand implementation to scale as soon as possible" [113].

In contrast to OST, other forms of drug dependence treatment have not usually been introduced in prison with HIV prevention as one of their objectives. Therefore, there is little data on their effectiveness as an HIV prevention strategy [113].

Nevertheless, good quality, appropriate and accessible treatment has the potential of improving prison security, as well as the health and social functioning of prisoners, and might reduce re-offending. Studies have demonstrated the importance of providing ongoing treatment and support and of meeting the individual needs of prisoners, including female prisoners, younger prisoners and prisoners from ethnic minorities [113]. Given that many prisoners have severe problems related to the use of illegal drugs, it would be unethical not to provide people in prison with access to a wide range of drug treatment options [119].

Therefore, WHO, UNODC and UNAIDS have recommended that, in addition to providing OST, prison authorities also provide a range of other drug dependence treatment options for prisoners with problematic drug use, in particular for other substances, such as amphetamine-type stimulants. However, because data on the effectiveness of these other forms of treatment as an HIV prevention strategy are lacking, they recommended that evaluations of their effectiveness in terms of reducing drug injecting and needle sharing should be undertaken [113].

While drug-free or abstinence-based treatment should be considered as a necessary element of comprehensive prison drug services, such programmes alone are insufficient to address the multiple health risks posed by injecting drug use and HIV transmission in prisons.

In some countries, including Cambodia, China, Indonesia, Laos, Malaysia, Myanmar, Thailand and Vietnam, people who use drugs can face coerced "treatment" and "rehabilitation" in compulsory drug detention centres, which results in many human rights abuses [31]. In many of these centres, the services provided are of poor quality and do not accord with either human rights or scientific principles. Treatment in these facilities takes the form of sanction rather than therapy, and relapse rates are very high [120]. These centres should be closed and replaced with drug treatment that works.

#### Other measures to reduce the demand for drugs

In addition to drug dependence treatment, other strategies to reduce the demand for drugs can also assist efforts to prevent HIV transmission in prisons. However, it is important to note from the outset that such efforts are unlikely to eliminate drug use in prisons. In fact, even prison systems that have devoted large financial resources to such efforts have not been able to eliminate drug use [113]. Therefore, such efforts cannot replace the other measures that we have described, but rather should be undertaken to complement them.

##### Provision of information on drugs and drug use

On its own, the provision of information on drugs and drug use has not been found to change drug use behaviour. However, substantial and correct information is necessary to make healthy choices, and all drug dependence programmes should include an education component [121].

##### Work, study and other activities

Research shows that one of the reasons why some prisoners take drugs when they are in prison is to combat boredom and alienation, and to promote relaxation [122]. This suggests a need for more purposeful activities in prisons. Providing prisoners with opportunities to work and/or study while in prison, or to take part in activities, such as sports, theatre and spiritual and cultural enhancement aimed at providing people with challenging and healthy ways to employ their time, can have a positive effect on risky behaviours, particularly when complemented by appropriate drug use prevention education (which might include both information and life skills provision).

##### Life skills education

Providing life skills education is also important. Life skills are the abilities for adaptive and positive behaviour that enable individuals to deal effectively with the demands and challenges of everyday life. These include: self-awareness, empathy, communication skills, interpersonal skills, decision-making skills, problem-solving skills, creative thinking, critical thinking, and coping with emotions and stress. Such personal and social competencies, together with appropriate information about drugs and drug use, help people make healthier choices.

##### Establishing so-called "drug-free" units

Another strategy to reduce the demand for drugs used by an increasing number of prison systems, mainly in resource-rich countries, is to establish so-called "drug-free" units. Typically, "drug-free" units or wings are separate living units within a prison that focus on limiting the availability of drugs, and are populated with prisoners who have voluntarily signed a contract promising to remain drug free. In some instances, they focus solely on drug interdiction through increased searching, while some systems provide a multi-faceted approach combining drug interdiction measures with treatment services.

"Drug-free" units could assist efforts to combat the spread of HIV in prison if they resulted in decreased drug use, particularly injecting drug use. There is some evidence from a small number of studies that "drug-free" units do indeed significantly reduce levels of drug use among residents in these units [113]. Such units appeal to a large number of prisoners, including prisoners who do not have any drug problems and want to live in a "drug-free" environment. However, the studies do not say anything about whether "drug-free" units appeal to, and are successful in retaining, the most problematic drug users, in particular prisoners who inject drugs. Currently, there is therefore no data on the effectiveness of drug-free units as an HIV prevention strategy [113].

#### Measures to reduce the supply of drugs

A broad range of search and seizure techniques and procedures can be used in an attempt to reduce the availability of drugs in prisons. These supply reduction measures include: random searches by security personnel; prison staff and visitor entry/exit screening and searches; drug detection dogs; closed-circuit monitoring; perimeter security measures (netting over exercise yards, higher internal fences to prevent projectiles, rapid response vehicles patrolling the prison perimeter); purchasing of goods from approved suppliers only; intelligence analysts at every institution; drug detection technologies (such as ion scanners and X-ray machines); modifications to the design and layout of visiting areas (use of fixed and low-level furniture); and drug testing (also called urinalysis).

Many prison systems, particularly in resource-rich countries, have placed considerable emphasis on these measures to reduce the supply of drugs. While such measures are not aimed at addressing HIV in prisons, they may result in unintended consequences for HIV (and HCV) prevention efforts. Drug interdiction measures may assist HIV prevention efforts by reducing the supply of drugs and injecting in prisons. At the same time, they could make such efforts more difficult.

For example, many resource-rich prison systems regularly or randomly test prisoners' urine for illegal drug use. Prisoners who are found to have consumed illegal drugs can face penalties. From a public health perspective, concerns have been raised that these programmes may increase, rather than decrease, prisoners' risk of HIV infection. There is evidence that implementing such programmes may contribute to reducing the demand for and use of cannabis in prisons [123,124].

However, such programmes seem to have little effect on the use of opiates [114,125]. In fact, there is evidence that a small number of people may switch to injectable drugs to avoid detection of cannabis use through drug testing [113]. Cannabis is traceable in urine for much longer (up to one month) than drugs administered by injecting, such as heroin and other opiates. Some prisoners choose to inject drugs rather than risk the penalties associated with smoking cannabis simply to minimize the risk of detection and punishment. Given the scarcity of sterile needles and the frequency of needle sharing in prison, the switch to injecting drugs may have serious health consequences for prisoners.

Generally, despite the fact that many prison systems make substantial investments in drug supply reduction measures, there is little solid and consistent empirical evidence available to confirm their efficacy in reducing levels of drug use. In particular, there is no evidence that these measures may lead to reduced HIV risk [113].

Prison systems facing resource constraints should therefore not implement costly measures, such as drug detection technologies and drug testing, that may use up a substantial amount of resources that could otherwise be used for managing HIV/AIDS in prisons. Instead, they should focus on the proven and cost-effective HIV prevention measures that we have described and on efforts to improve prison conditions and working conditions and pay for prison staff, without whom other drug supply reduction strategies are unlikely to be successful [113,122].

#### Other measures

##### Detection and treatment of sexually transmitted infections

Early detection and treatment of sexually transmitted infections (STIs) is important because these infections increase the chances of an individual acquiring and transmitting HIV [122].

##### Post-exposure prophylaxis

There is evidence from studies in the community that provision of antiretroviral drugs to prevent HIV infection after unanticipated sexual exposure might be beneficial [126]. This has resulted in recommendations that post-exposure prophylaxis (PEP) be made available to persons seeking care less than 72 hours after exposure to blood, genital secretions or other potentially infectious body fluids of a person known to be HIV infected when that exposure represents a substantial risk for transmission. PEP refers to a set of services to prevent the infection from developing in the exposed person. These include: first aid care; counselling and risk assessment; HIV testing following informed consent; and depending on risk assessment, the provision of short-term (28 days) antiretroviral drugs. If indicated, antiretroviral drugs should be initiated as soon as possible after exposure [126].

Recommendations have also been formulated for other scenarios in which PEP may be offered [127]. In particular, use of PEP has been widely encouraged for victims of sexual assault [128-130].

In the first documented use of PEP in the prison setting anywhere in the world, 46 prisoners in Australia were offered PEP, and 34 elected to receive it, but only eight completed the full PEP course. The study concluded that PEP administration in prisons is feasible, but that special consideration of prison circumstances is necessary to ensure accurate risk assessment, consideration of ongoing risk behaviours, prompt initiation of therapy, good compliance and adequate follow up [131].

WHO, UNODC and UNAIDS have recommended that prison systems should make PEP available in cases in which it could reduce the risk for HIV transmission after exposure to HIV. Specific guidelines for the use of PEP in prisons should be developed by correctional health services to improve the administration of PEP in the prison setting [101].

##### Controlling the spread of TB

TB poses a substantial danger to the health of all prisoners, prison staff and the community outside prisons. Prisoners living with HIV are at particular risk. HIV infection is the most important risk factor for the development of TB, and TB is the main cause of death among people living with HIV. For these reasons, in addition to improving conditions in prisons that fuel the spread of TB, prisons must develop and implement comprehensive TB control programmes, which should be coordinated with or integrated in national TB control programmes and work closely with the HIV programme [121].

##### Hepatitis B vaccination

Hepatitis B is easily spread in prisons. In contrast to HIV, the risk of infection can be reduced through the administration of a vaccine. All staff and prisoners should have easy access to free hepatitis B vaccination. In addition, consideration should be given to providing hepatitis A vaccination to prisoners at risk [121].

##### Hepatitis C prevention

In addition to contributing to reduced risk of HIV transmission in prisons, most of the measures just described also contribute to reducing the risk of hepatitis C virus (HCV) transmission. However, HCV is much more easily spread than HIV, including through sharing of shavers and toothbrushes, as well as through tattooing and body piercing [121]. It is therefore important that prisons make information available to all prisoners and staff about the risks of HCV transmission in prison, and educate them about the ways to reduce that risk. In addition, shavers and toothbrushes should be made available to prisoners so that they do not have to share them with fellow prisoners; and prisons should consider implementing measures to reduce the spread of HCV through tattooing and body piercing, such as making sterile tattooing equipment available to prisoners [121].

#### Protecting staff

Protection of staff from infectious diseases is a duty and also part of good prison management. High rates of HIV and other infectious diseases in prisons make them more stressful places in which to work. High rates of staff turnover, whether due to ill health or lack of job satisfaction have a major impact on the management of prisons [121].

It is essential that staff receive initial and ongoing training to enable them to do their duties in a healthy and safe manner, and to feel secure themselves and be able to give prisoners appropriate guidance and support. This training should enable them to anticipate and manage situations in which they may be exposed to HIV or hepatitis. Staff should also be trained in the safe provision of first aid.

When on duty, relevant prison staff should have access to personal protective equipment, such as latex gloves, masks for use in mouth-to-mouth resuscitation, protective eyewear, soap, and mirrors for use in searching. Staff should also have free access to hepatitis B vaccination.

Safe work procedures should be developed, including searching procedures. Post-exposure procedures also must be in place. The procedures should address immediate action, follow-up action, record keeping and confidentiality. Finally, staff should have access to appropriate professional counselling and follow-up services, including PEP, after possible and definite exposures to blood and body fluids. Finally, ample space, adequate lighting and optimum staffing levels are important to ensure safe work practices, and measures are required to improve the general work conditions of prison staff.

In contrast, it is *not *important to know the HIV status of prisoners (and prison staff), and all must be handled equally - as if they were HIV positive, both for safety reasons and in order to avoid discrimination [121].

### Providing treatment, care and support, including provision of antiretroviral treatment for HIV

In addition to providing comprehensive HIV prevention programmes, national governments have a responsibility to provide prisoners with treatment, care and support equivalent to that available to other members of the community.

Health in prison is a right guaranteed in international law, as well as in international rules, guidelines and covenants [95]. The right to health includes the right to medical treatment and to preventive measures and to standards of health care equivalent to those available in the community. As it was stated in April 1996 by UNAIDS to the United Nations Commission on Human Rights at its 52^nd ^session [132]:

HIV/AIDS in prisons remains a difficult and controversial subject.... Often there are not enough resources to provide basic health care in prisons, much less HIV/AIDS programmes. Yet the situation is an urgent one. It involves the rights to health, security of the person, equality before the law and freedom from inhuman and degrading treatment.... With regard to effective HIV/AIDS prevention and care programmes, prisoners have a right to be provided the basic standard of medical care available in the community.

#### Effective HIV treatment in prison settings

The right to medical care in prisons includes the provision of antiretroviral therapy (ART) in the context of comprehensive HIV care [133]. The advent of combination ART has significantly decreased mortality due to HIV infection and AIDS in countries around the world where ART has become accessible. There has been a parallel decrease in the mortality rate among incarcerated individuals in prison systems in those countries.

Providing access to ART for those in need in prisons is a challenge, but it is necessary and feasible. Studies have documented that, when provided with care and access to medications, prisoners respond well to antiretroviral treatment [134]. The right to enjoyment of the highest attainable standard of physical and mental health, in concert with the principle of equivalence, dictates that prisoners should have access to the same standard of care available to people outside prisons.

As ART has increasingly become available in developing countries and countries in transition, and as countries are moving towards the goal of universal access to HIV prevention, treatment, care and support, it is critical to ensure that treatment also becomes available to all prisoners who need it. Ensuring continuity of care from the community to the prison and back to the community, as well as continuity of care within the prison system, is a fundamental component of successful treatment scale-up efforts. Treatment discontinuation for short or long periods of time may happen upon arrest and detention in police cells, within the prison system when prisoners are transferred to other facilities or have to appear in court, and upon release. Each of these situations should be addressed and mechanisms established to ensure uninterrupted ART [135,136]. Particular attention should be devoted to discharge planning and linkage to community aftercare.

In addition, the following actions will facilitate continuity of treatment [133]:

• Prison departments must have a place within the national HIV/AIDS coordinating committees, and prison issues need to be part of the agreed HIV/AIDS action framework and country-level monitoring and evaluation system.

• Prison departments need to be involved in all aspects of treatment scale up, from applications for funding (to ensure that funds are specifically earmarked for prisons), to development, implementation, and monitoring and evaluation of treatment roll-out plans.

• The ministry responsible for health and the ministry responsible for the prison system should collaborate closely, recognizing that prison health is public health.

• Policies or guidelines should be developed specifying that people with HIV or AIDS are allowed to keep their HIV medication upon them, or are to be provided with their medication, upon arrest and incarceration, and at any time that they are transferred within the system or to court hearings. Police and prison staff need to be educated about the importance of continuity of treatment.

#### Prison healthcare: the need for increased funding and a new model

Health services in prison settings are most often sub-standard and underfunded, and short of staff, of essential medications, of equipment and of appropriate infrastructures. Often, health services in prison settings work in complete isolation from the general healthcare system, hampering the quality of healthcare and making continuity of care a challenge. HIV/AIDS, HCV and TB have exacerbated existing problems in healthcare provision in prisons. Prison healthcare budgets must reflect the growing needs of the prison population. Prison healthcare should be recognized as an integral part of the public health sector, and evolve from its present reactive "sick call" model into a proactive system that emphasizes early disease detection and treatment, health promotion and disease prevention.

There is a need for a public health infrastructure to fulfill the core functions of public health services within prisons, i.e., to: assess the health status of prisoners; have an effective surveillance system for infectious and chronic diseases; undertake health promotion efforts; have coordinated actions to prevent diseases and injuries; protect the health of prisoners; and evaluate the effectiveness, accessibility and quality of health services [137]. Addressing prisoners' health needs will contribute to the prisoner's rehabilitation and successful reintegration into the community [121].

#### Transferring control of prison health

In the longer term, transferring control of prison health to public health authorities could have a positive impact on HIV/AIDS care in prison [138]. In the vast majority of prison systems in the world, healthcare is provided by the same ministry or department responsible for prison administration, not by the ministry or department responsible for healthcare. Prisons were not designed and are generally not equipped to deal with prisoners infected with chronic, potentially fatal diseases, such as HIV/AIDS, hepatitis and tuberculosis. They do not have adequate staffing levels, adequate staff training or adequate equipment to meet the health needs of prisoners suffering from these diseases.

The authority and influence of prison authorities may compromise healthcare professionals' ethical obligations. Trust and confidence are crucial to an effective, ethical relationship between patient and healthcare provider. When health services for prisoners are "captured" within, or subservient to, the prison administration, it is unlikely that prisoners will trust or have confidence in the healthcare providers. This lack of trust contributes to sub-standard healthcare for prisoners [95].

Experience in a range of prison systems has shown that healthcare in prisons can be delivered more effectively by public health authorities than by prison management. This has the advantage of strengthening the link between health in the community and health in prisons [136,138,139]. Some countries have already introduced such a change in prison health administration. Norway was one of the first. In France, where prison health was transferred to the Ministry of Health in 1994, a positive impact is already evident [139]. Each prison in France is twinned with a public hospital.

#### Special attention should be given to women prisoners

As women prisoners are fewer than males, the health services provided for women are sometimes minimal or second rate. With the advent of HIV/AIDS, a new problem has arisen for women prisoners. Women prisoners need the same preventive measures and the same level of care, treatment and support as male prisoners. Pregnant prisoners need access to the full range of prevention of mother to child transmission interventions. In addition, there is a need for initiatives that acknowledge that the problems encountered by women in the correctional environment often reflect, and are augmented by, their vulnerability and the abuse many of them have suffered outside prison. The task of protecting women prisoners from HIV transmission and of providing those living with HIV with care, treatment and support therefore presents different - and sometimes greater - challenges than that of dealing with HIV infection in male prisoners [140].

## Conclusions

Much can be done to address the problems linked to HIV and other infections in prisons by taking action in the areas that we have outlined. In the medium- and longer-term, however, it will be essential to take action to improve prison conditions and reduce overcrowding. Prison conditions are integrally linked to prison health, and have the potential to affect the health of prisoners in positive or negative ways.

Sub-standard living conditions and overcrowding, as described at the beginning of this article, can increase the risk of HIV transmission among prisoners by promoting and encouraging drug use in response to boredom or stress (most often involving unsafe injecting practices) and by enabling prison violence, fighting, bullying, sexual coercion and rape. They can also have a negative impact on the health of prisoners living with HIV by: increasing their exposure to infectious diseases, such as TB and hepatitis; housing them in unhygienic and unsanitary environments; confining them in spaces that do not meet the minimum requirements for size, natural lighting and ventilation; limiting access to open-air and to educational, social or work activities; and failing to provide them with access to proper healthcare, diet, nutrition and/or clean drinking water, and basic hygiene [121]. A comprehensive programme of prison reform based on the international human rights standards would do much to improve sub-standard living conditions and, ultimately, to reduce the spread of HIV.

In addition, taking measures to reduce prison populations is essential. In the short term, overcrowding can be reduced by amnesties, reviewing the legality of detention status so that those held unlawfully can be released, and removing groups inappropriately held, such as those prisoners with mental disorders.

In the medium and long term, there are two potential solutions to overcrowding: increasing the capacity of the prison system; or reducing the number of prisoners. The first solution is very costly, and many countries do not have the additional financial resources required to expand their prison systems in ways that respect basic human rights standards or could put those resources to better use. Reducing imprisonment and pre-trial detention is the better solution. Prison should only be used as a place of last resort. In all other cases, alternatives to custody should be used. A range of community-based pre-trial and sentencing options and programmes of supervised early release can help to ensure that prison is used as a last resort and for the shortest time. A good strategy is to adopt official government targets for reducing prison overcrowding [121].

The overuse of incarceration of people who use drugs is of particular concern. In many countries, a significant percentage of the prison population is comprised of individuals who are convicted of offences directly related to their own drug use (i.e., those incarcerated for the possession of small amounts of drugs for personal use, and those convicted of petty crimes specifically to support drug habits). The incarceration of significant numbers of people who use drugs increases the likelihood of drug use inside prisons, as well as unsafe injecting practices and the risk of transmission of HIV and other blood-borne diseases.

Many of the problems created by HIV infection and by drug use in prisons could be reduced if alternatives to imprisonment are implemented, particularly in the context of drug-related crimes. As early as 1987, the World Health Organization, in a statement from the first Consultation on Prevention and Control of AIDS in Prisons, said that "[g]overnments may ... wish to review their penal admission policies, particularly where drug abusers are concerned, in the light of the AIDS epidemic and its impact on prisons" [141].

## Competing interests

The authors declare that they have no competing interests.

## Authors' contributions

RJ took primary responsibility for the writing of all sections of the paper with the exception of the first section (Forgotten prisoners: global crisis of conditions in detention), which was written by MN. MD was involved in all discussions of the article and provided extensive comments that were integrated in the final version of the article. All authors read and approved the final manuscript.

## Authors' information

MN is the director of the Ludwig Boltzmann Institute of Human Rights in Vienna and a professor for international human rights protection at the University of Vienna. He served as a member of the United Nations Working Group on Enforced or Involuntary Disappearances and as the UN Special Rapporteur on Torture and Other Cruel, Inhuman or Degrading Treatment.

RJ is one of the co-founders of the Canadian HIV/AIDS Legal Network and was its Executive Director from 1998 to November 2004. Since December 2004, he has worked as a consultant on HIV/AIDS, health, policy and human rights in eastern Europe, central Asia, Africa and Canada. He is the author of many reports and more than 100 articles on legal, ethical and human rights issues related to HIV, including four papers in the World Health Organization's "Evidence for Action" series on the evidence of interventions to address HIV in prisons. From 1992 to 1994, Ralf was the coordinator of Canada's Expert Committee on AIDS in Prisons.

MD is the technical advisor for drugs and HIV to the Association of Caribbean Heads of Corrections and Prison Services (ACHCPS), the umbrella body of the heads of CARICOM correctional institutions. He is also technical advisor to the CARICOM Secretariat for the same. Over the past 10 years, he has done extensive research and training in Caribbean prisons on issues related to illicit drug use and HIV.
